# Intelligence May Moderate the Cognitive Profile of Patients with ASD

**DOI:** 10.1371/journal.pone.0138698

**Published:** 2015-10-07

**Authors:** Nanda Rommelse, Ilse Langerak, Jolanda van der Meer, Yvette de Bruijn, Wouter Staal, Anoek Oerlemans, Jan Buitelaar

**Affiliations:** 1 Radboud University Nijmegen Medical Centre, Nijmegen, Donders Institute for Brain, Cognition and Behavior, Department of Psychiatry, Nijmegen, The Netherlands; 2 Karakter, Child and Adolescent Psychiatry University Centre Nijmegen, Nijmegen, the Netherlands; 3 Radboud University Nijmegen Medical Centre, Nijmegen, Donders Institute for Brain, Cognition and Behavior, Department of Cognitive Neuroscience, Nijmegen, The Netherlands; University College London, UNITED KINGDOM

## Abstract

**Background:**

The intelligence of individuals with Autism Spectrum Disorder (ASD) varies considerably. The pattern of cognitive deficits associated with ASD may differ depending on intelligence. We aimed to study the absolute and relative severity of cognitive deficits in participants with ASD in relation to IQ.

**Methods:**

A total of 274 children (*M* age = 12.1, 68.6% boys) participated: 30 ASD and 22 controls in the below average Intelligence Quotient (IQ) group (IQ<85), 57 ASD and 54 controls in the average IQ group (85<IQ<115) and 41 ASD and 70 controls in the above average IQ group (IQ>115). Matching for age, sex, Full Scale IQ (FSIQ), Verbal IQ (VIQ), Performance IQ (PIQ) and VIQ-PIQ difference was performed. Speed and accuracy of social cognition, executive functioning, visual pattern recognition and basic processing speed were examined per domain and as a composite score.

**Results:**

The composite score revealed a trend significant IQ by ASD interaction (significant when excluding the average IQ group). In *absolute* terms, participants with below average IQs performed poorest (regardless of diagnosis). However, in *relative* terms, above average intelligent participants with ASD showed the most substantial cognitive problems (particularly for social cognition, visual pattern recognition and verbal working memory) since this group differed significantly from the IQ-matched control group (*p* < .001), whereas this was not the case for below-average intelligence participants with ASD (p = .57).

**Conclusions:**

In *relative* terms, cognitive deficits appear somewhat more severe in individuals with ASD and above average IQs compared to the below average IQ patients with ASD. Even though high IQ ASD individuals enjoy a certain protection from their higher IQ, they clearly demonstrate cognitive impairments that may be targeted in clinical assessment and treatment. Conversely, even though in *absolute* terms ASD patients with below average IQs were clearly more impaired than ASD patients with average to above average IQs, the differences in cognitive functioning between participants with and without ASD on the lower end of the IQ spectrum were less pronounced. Clinically this may imply that cognitive assessment and training of cognitive skills in below average intelligent children with ASD may be a less fruitful endeavour. These findings tentatively suggest that intelligence may act as a moderator in the cognitive presentation of ASD, with qualitatively different cognitive processes affected in patients at the high and low end of the IQ spectrum.

## Introduction

Autism Spectrum Disorder (ASD) is a severely impairing and clinically heterogeneous disorder characterized by impairments in social interactions and social communication across multiple contexts and by restricted interests and/or repetitive behaviour [1]. One key factor that may influence phenotypic variance in the ASD spectrum is intelligence. Intelligence (intelligence quotient, IQ) is a strong predictor of outcome in terms of school, work and social functioning, both in ASD [2] and non-ASD populations [3]. A substantial proportion of individuals with ASD suffers from severe to mild Intellectual Disability (ID) [4–6]. This is most likely explained by the substantial shared genetic etiology of both disorders [7, 8], although evidence also exists for unique genetic factors influencing ASD and IQ [9]. In addition, certain prenatal factors have been found to influence both disorders, such as the prenatal exposure to infections [10–12] and medications as thalidomide and valproate [13–16] and preterm delivery [17, 18]. Nevertheless, average to above average IQs and even giftedness are also present in individuals with ASD, though the exact percentage is unclear [19,20].

It has been hypothesized that IQ scores may not be directly comparable between ASD and non-ASD patients. That is, IQ scores may reflect different underlying processes in ASD patients and non-patients. For instance, several lines of research indicate that general intelligence (‘g’), the positive correlation found between all mental tests, reflecting the tendency to perform equally well across various cognitive domains [21], is strongly related to the basic speed of information processing [22, 23]. However, in contrast to what would be expected based on their IQ scores, data suggest that the speed of basic information processing may not necessarily be impaired in ASD patients [24], or not correlated to IQ [25], suggesting involvement of partially distinct factors influencing IQ in ASD patients versus controls [24]. This hypothesis is further supported by the increased frequency of savant skills in ASD patients [26], and disharmonic IQ profiles [27]. Even in the presence of comparable performances, ASD patients may recruit different areas of their brain whilst performing the task [28]. These findings suggest that similar IQ scores may reflect different underlying processes in ASD patients and controls, both in the lower and higher ends of the IQ spectrum. This knowledge should be taken into account when examining ASD across the IQ distribution.

It is well known that IQ influences the phenotypic presentation of ASD. This is most prominently the case for restrictive repetitive behaviors [29] and challenging behaviors [30]. However, it is largely unknown to what extent IQ influences the cognitive deficits often seen in individuals with ASD. Cognitive domains often reported to be affected in ASD include social cognition, executive functioning, basic processing speed, language and sensory processing [31]. On the one hand it may be hypothesized that only quantitative (i.e. severity) cognitive differences exist in ASD patients with a low versus above average IQ profiles, as has been previously documented for executive skills [32, 33]. Intelligence can then best be seen as a severity marker, but not as a moderator of clinical presentation. On the other hand, intelligence may act as a moderator in the cognitive presentation of ASD, with qualitatively different underlying affected cognitive processes in individuals with ASD with above and below average IQs. More insight into whether similar or different cognitive processes underlie ASD at the low and high ends of range of intelligence may have implications for clinical assessment and treatment of ASD individuals. In addition, many studies of ASD use only participants with average IQs and if an intelligence moderates cognitive deficits associated with ASD, the results of these studies will not generalize to individuals with below or above average IQs.

Therefore, the current study set out to examine the absolute and relative severity of cognitive deficits in participants with ASD in relation to IQ. In order to do so, one group of below average (70<IQ<85), one group of average (85<IQ<115) and one group of above average (IQ>115) IQ ASD patients were included carefully matched for FSIQ, VIQ (verbal IQ), PIQ (performance IQ), VIQ-PIQ discrepancy, age, and gender with controls. A battery of cognitive tasks was administered tapping into several key cognitive domains relevant to ASD, namely social cognition (SC), executive functioning (EF), visual pattern recognition [34–37] and basic processing speed [38–40]. The main effects of diagnosis and IQ as well as the interaction diagnosis by group were examined for all domains.

## Method

### Participants

The study protocol was approved by the local medical-ethics committee (‘Commissie Mensgebonden Onderzoek (CMO) Arnhem-Nijmegen’). Written informed consent was obtained from parents and children before testing. Children with the diagnosis of DSM-IV defined ASD (Autism, AS or PDD-NOS) [41] were recruited as part of the Biological Origins of Autism (BOA) study at Karakter Child and Adolescent Psychiatry, University Medical Center Nijmegen, the Netherlands, which aims to examine the genetic, biochemical and cognitive origins of ASD. All children were similarly screened for autism spectrum disorders using the parent Social Communication Questionnaire (SCQ) [42]. Clinical diagnosis of ASD was confirmed using the Autism Diagnostic Interview- revised (ADI-R) by a certified clinician [43]. Children were considered ASD probands if they were clinically diagnosed according to the DSM-IV algorithm and if they fulfilled ADI-R criteria. No Autism Diagnostic Observation Schedule (ADOS) was performed due to time constraints.

Control children were recruited via public schools and information leaflets that were sent to families living in the same geographical regions as the participating ASD children as part of the BOA project or the SPIDER project (Schoolkids Project Interrelating DNA and Endophenotype Research) [34, 44]. Controls were required to have no formal or suspected ASD diagnosis or related psychiatric disorder (such as ADHD) and had to score below clinical cut-off (<12) on the SCQ.

All children were between the ages of 6 and 21 and were of European Caucasian descent. Participants were excluded if they had a diagnosis of epilepsy, brain disorders or known genetic disorders, such as Down-syndrome or Fragile-X-syndrome. FSIQ was estimated using four subtests of the Dutch version Wechsler Intelligence Scale for children (WISC-III) or Wechsler Adult Intelligence Scale (WAIS-III): Similarities, Block Design, Picture Completion and Vocabulary [45]. These selected subtests are known to correlate between .90-.95 with the Full-scale IQ [46]. For children older than 16 years, the WAIS-III was administered [47]. VIQ was estimated based on Vocabulary and Similarities; PIQ was estimated based on Block Patterns and Picture Completion.

A subset of the complete sample was used for this study. The selection of this sample was performed in the following steps. In the first step, we selected the smallest group for this study, namely all available controls with an IQ <85 (below average IQ group). We then matched carefully on a group level the below average IQ ASD group regarding FSIQ, VIQ, PIQ, VIQ-PIQ discrepancy, age, and gender. Thereafter, we selected the above average IQ (IQ>115) ASD group and matched the group regarding age with both below average IQ groups. Complete group matching for gender was not feasible, since above average IQ scores were predominantly obtained by males with ASD. Then we selected an above average IQ control group matched regarding all parameters with the above average IQ ASD group. Lastly, both average IQ (85<IQ<115) groups were selected matched regarding age with all other groups and regarding proportion males/females in between the low and above average IQ groups. The matching procedure made the best use of the available data. This resulted in a total of 274 children in this study: 30 ASD and 22 controls in the below average IQ group, 57 ASD and 54 controls in the average IQ group and 41 ASD and 70 controls in the above average IQ group. Ninety percent of the sample was between 8 and 16 years old. Despite every effort to match the groups on all characteristics, the above average IQ group contained slightly more males than the low and average IQ groups (*F* (2, 273) = 7.49, *p* = .001) (Table 1). In addition, because the number of below average IQ controls was relatively small in comparison to above average IQ controls, the average IQ scores of the complete control sample were somewhat higher (*p*’s < .03) (FSIQ = 108.4, VIQ = 108.1, PIQ = 109.3) than the average IQ scores of the complete ASD sample (FSIQ = 103.2, VIQ = 102.7, PIQ = 103.9).

**Table 1:** Participant characteristics.

	Below average (70<IQ ≤85)			Average (85<IQ<115)			Above average (IQ ≥ 115)			Low versus normal versus above average IQ	ASD versus control
	ASD	Control	Contrasts	ASD	Control	Contrasts	ASD	Control	Contrasts		
***N***	30	22		57	54		41	70			
**Age** *(M*, *SD)*	12.1 (3.0)	12.8 (3.4)	n.s.	12.2 (2.4)	12.2 (2.7)	n.s.	11.4 (3.1)	12.2 (3.6)	n.s.	n.s.	n.s.
**Sex** *(N♂*, *%)*	17 (56.7)	10 (45.5)	n.s.	37 (64.9)	35 (64.8)	n.s.	35 (85.4)	54 (77.1)	n.s.	Low = Normal<High	n.s.
**SCQ** ^a^ *(M*, *SD)*	18.8 (7.0)	4.1 (2.9)	ASD>Control	18.9 (6.6)	2.7 (2.1)	ASD>Control	16.9 (6.1)	3.0 (2.5)	ASD>Control	n.s.	Control<ASD
**FSIQ** ^b^ *(M*, *SD)*	79.3 (6.2)	79.8 (6.1)	n.s.	102.4 (7.4)	103.0 (7.3)	n.s.	121.7 (5.4)	121.7 (6.0)	n.s.	Low<Normal<High	Control>ASD
**VIQ** ^c^ *(M*, *SD)*	80.6 (10.1)	82.7 (10.0)	n.s.	101.9 (7.4)	102.2 (8.8)	n.s.	119.9 (9.1)	120.6 (9.5)	n.s.	Low<Normal<High	Control>ASD
**PIQ** ^d^ *(M*, *SD)*	76.7 (8.0)	75.2 (8.0)	n.s.	103.0 (11.4)	103.9 (9.8)	n.s.	125.1 (11.4)	124.2 (10.3)	n.s.	Low<Normal<High	Control>ASD
**VIQ-PIQ** ^e^ *(M*, *SD)*	3.9 (13.2)	7.5 (13.1)	n.s.	-1.07 (11.5)	-1.7 (10.9)	n.s.	-5.3 (17.3)	-3.6 (15.4)	n.s.	Low>Normal = High	n.s.

Note.

^a^ Social Communication Questionnaire.

^b^ Total IQ based on four subtests (Similarities, Block Patterns and Picture Completion, Vocabulary/Arithmetic).

^c^ Verbal IQ based on two subtests (Vocabulary/Arithmetic and Similarities).

^d^ Performance IQ based on two subtests (Block Patterns and Picture Completion).

^e^ Difference between verbal IQ and performance IQ; positive outcome means in favor of verbal IQ, negative outcome means in favor of performance IQ.

### Instruments

In total, seven tasks were selected from the Amsterdam Neuropsychological Tasks (ANT) program [48] and one subtest (Digit Span) of the Wechsler Intelligence Scale [45, 47] to study social cognition (SC), executive functioning (EF), visual pattern recognition [34] and basic processing speed. The ANT is a computer-aided assessment battery that allows for the systematic evaluation of information processing capacities and has been proven to be a sensitive and valid tool in research into autism-related disorders. Test–retest reliability and validity of the ANT-tasks are satisfactory and have been described and illustrated elsewhere [49]. A full description of each task is described elsewhere [34, 44] and a summary description is provided in the Table 2. Each computer task contained an instruction trial where the examiner provided a typical item of the task, and a separate practice session. If necessary the instruction was repeated. All participants were able to perform the training items before testing. Digit Span was administered following manual guidelines [45, 47]. For all ANT tasks the main outcome variables were speed (mean reaction time) and accuracy (number of errors) of responses. Missing data varied between 1.1% (3/274) for BPS and 18.2% (50/274) for affective prosody (see Table 3) and were not replaced. This did not affect group matching. Ceiling effects regarding accuracy occurred on the face recognition task (N = 24 [8.8%] made no errors); no ceiling effects on other tasks were present.

**Table 2:** Description of the cognitive measures.

Cognitive domain	Task	Description	Dependent variable(s)
**Basic processing speed**			
	Baseline speed^a^	A fixation cross in the centre of a computer screen changed unpredictably into a white square. Participants were required to press a key when the white square emerged.	Mean reaction time (ms) and variability (SD of reaction time in ms)^c^
**Social cognition**			
Facial Emotion Recognition	Facial Emotion Recognition^a^	Children were asked to judge whether the presented photograph showed the target emotion (happiness, sadness, anger or fear) or a non-target expression (1 of 8 different emotions: happiness, sadness, anger, fear, disgust, surprise, shame, contempt), by pressing a mouse button.	Mean reaction time (ms) and % errors on four emotions
Face recognition	Face recognition^a^	Children were asked to identify a neutral target face in a display set that consisted of four neutral faces.	Mean reaction time (ms) and % errors
Prosody	Prosody^a^	Stimuli consisted of spoken sentences with a neutral content, presented through a headphone. Sentences were spoken in a happy, sad, angry or frightened manner. Children were asked to verbally identify the emotion with which the sentence was spoken out.	Mean reaction time (ms) and % errors
**Executive functioning**			
Inhibition	Response Organization Objects^a^	Stimuli consisted of a horizontal gray bar with green or red moving squares. The task consisted of three blocks. In the first block, the moving square was colored green, and compatible responses were required. In the second block, the moving square was colored red, and incompatible responses were required. In the third block, the color of the moving square alternated randomly between green and red, and both compatible and incompatible responses were required.	Difference in % errors or mean reaction time (ms) between Block 1 (compatible trials only) and Block 2 (incompatible trials only).
Cognitive flexibility	Response Organization Objects^a^	See above	Difference in % errors or mean reaction time (ms) between compatible trials in Block 1 (compatible trials only) and compatible trials in Block 3 (mixed compatible and incompatible trials)
Visuo-Spatial Working Memory	Spatial Temporal Span^a^	Nine circles symmetrically organized in a square (3 by 3). Repeating the identified circles in the opposite order.	Number of correct targets in reversed (backward) order
Verbal Working Memory	Digit Span^b^	Repeating the numbers in the opposite order.	Number of correct reproduced sequences in reversed (backward) order
**Visual pattern recognition**			
	Pattern recognition task^a^	The stimulus was a predefined target pattern of 3 red and 6 white squares in a 3x3 matrix. After memorization of this target pattern children were asked to detect the target stimulus in a signal consisted of four patterns.	Mean reaction time (ms) and % errors

Note.

^a^ Amsterdam Neuropsychological Test (ANT) battery. For a full description of these tasks, please see (Oerlemans *et al*., 2013).

^b^ Wechsler Intelligence Scales (Wechsler, 2000, 2002).

^c^ Given the highly basic level of this task, virtually no errors are made by participants. The main indices are mean reaction time as well as the variability of reaction times. Both indices are correlated but also convey distinct underlying processes and are influenced by partly distinct etiological factors (see for instance). Therefore, both indices were included in the analyses.

**Table 3:** Means and standard deviations of the unstandardized cognitive task variables for below average, average and above average intelligent ASD and control children.

	Below average IQ	Below average IQ	Average IQ	Average IQ	Above average IQ	Above average IQ	*F*-, *p*- & *η*_*p*_^*2*^-values for univariate comparisons	*F*-, *p*- & *η*_*p*_^*2*^-values for univariate comparisons	*F*-, *p*- & *η*_*p*_^*2*^-values for univariate comparisons
	ASD	Control	ASD	Control	ASD	Control	IQ	Diagnosis	IQ x Diagnosis
**COMPOSITE SCORE**									
- standardized z-score	-0.34 (0.59)	-0.23 (0.57)	0.03 (0.50)	0.25 (0.45)	-0.06 (0.61)	0.48 (0.40)	**23.34/ < .001/.20**	**6.35/.01/.03**	2.11/.12/.01
**SOCIAL COGNITION**									
Face recognition n/N	29/30	14/22	56/57	54/54	40/41	49/70	*F/p/ η*_*p*_^***2***^	*F/p/ η*_*p*_^***2***^	*F/p/ η*_*p*_^***2***^
- MRT (*M*, *SD*)	1878.3 (581.7)	1541.9 (294.9)	1759.2 (556.3)	1608.9 (594.5)	2073.4 (648.0)	1504.2 (460.9)	3.57/.03/.04	**11.82/ < .001/ .05**	1.30/ .27/ .02
- % errors (*M*, *SD*)	21.0 (15.5)	14.8 (10.4)	16.9 (21.0)	10.2 (9.7)	18.3 (20.7)	9.8 (10.3)	**10.42/ < .001/.09**	1.89/ .17/ .02	0.58/ .56/ .01
Facial emotion recognition n/N	27/30	20/22	53/57	54/54	38/41	58/70			
- MRT (*M*, *SD*)	1038.5 (249.7)	947.5 (188.1)	935.7 (231.6)	913.1 (227.7)	1049.8 (219.0)	905.6 (179.6)	**5.70/ .004/.06**	5.54/ .019/.03	0.44/ .65/ .01
- % errors (*M*, *SD*)	17.8 (12.2)	13.9 (7.2)	15.2 (13.6)	11.5 (6.3)	16.2 (15.1)	11.7 (7.2)	**5.58/ .004/.07**	2.57/ .11/.01	0.39/ .68/.01
Affective prosody n/N	25/30	15/22	54/57	46/54	38/41	45/70			
- MRT (*M*, *SD*)	3646.8 (534.9)	3509.9 (598.9)	3505.5 (480.5)	3132.1 (705.5)	3500.1 (542.3)	3056.9 (692.1)	**6.89/ .001/.08**	4.66/ .032/.02	1.00/ .37/.01
- % errors (*M*, *SD*)	29.6 (11.4)	33.3 (14.6)	23.8 (9.2)	21.9 (10.7)	23.6 (9.8)	24.2 (11.2)	**9.34/ < .001/.10**	0.32/ .57/ < .01	1.72/ .18/.02
**EXECUTIVE FUNCTIONING**									
Inhibition n/N	28/30	22/22	56/57	53/54	40/41	70/70			
- MRT (*M*, *SD*)	204.6 (162.2)	155.5 (117.6)	144.9 (143.5)	114.0 (82.6)	157.5 (137.5)	98.1 (75.2)	**8.09/ < .001/.09**	**6.37/ .012/.02**	0.24/ .79/ < .01
- % errors (*M*, *SD*)	1.8 (5.2)	2.9 (4.5)	2.6 (4.5)	2.7 (4.1)	1.9 (4.3)	1.4 (4.1)	0.74/ .48/.01	0.09/ .76/ < .01	0.77/ .47/.01
Cognitive flexibility n/N	27/30	22/22	56/57	53/54	40/41	70/70			
- MRT (*M*, *SD*)	379.7 (216.5)	433.0 (280.3)	343.0 (252.9)	304.0 (183.6)	368.5 (237.7)	301.4 (150.1)	**3.93/ .021/.06**	0.01/ .94/ < .01	0.61/ .54/ < .01
- % errors (*M*, *SD*)	11.3 (12.4)	10.2 (10.7)	7.7 (8.4)	7.1 (7.7)	5.5 (7.7)	7.8 (8.6)	1.65/ .20/.05	0.04/ .85/ < .01	0.66/ .52/ < .01
Verbal working memory n/N	27/30	22/22	55/57	54/54	40/41	69/70			
- N correct (*M*, *SD*)	4.4 (1.5)	5.1 (1.3)	5.3 (1.8)	5.8 (1.7)	5.0 (2.1)	6.2 (2.4)	**5.25/ .006/.06**	**12.44/ < .001/.04**	0.42/ .66/.02
Visual working memory n/N	30/30	21/22	55/57	51/54	40/41	68/70			
- N correct (*M*, *SD*)	36.6 (21.6)	45.1 (16.2)	49.2 (24.0)	54.8 (18.7)	56.1 (20.2)	60.7 (19.3)	**17.93/ < .001/.12**	3.94/ .048/.01	0.11/ .90/.01
**VISUAL PATTERN RECOGNITION**									
Visual pattern recognition *n/N*	27/30	14/22	55/57	53/54	39/41	50/70			
- MRT (*M*, *SD*)	1701.7 (549.2)	1528.3 (392.1)	1524.0 (409.0)	1451.8 (458.0)	1666.1 (532.6)	1255.0 (285.5)	**7.14/ .001/.08**	4.76/ .03/.04	1.72/ .18/.02
- % errors (*M*, *SD*)	14.4 (11.0)	15.1 (9.4)	10.3 (8.3)	8.8 (6.0)	8.2 (5.5)	7.5 (5.7)	**10.41/ < .001/.10**	0.01/ .91/ < .01	0.65/ .53/.01
**BASIC PROCESSING SPEED**									
Basic processing speed *n/N*	29/30	22/22	57/57	53/54	40/41	70/70			
- MRT (*M*, *SD*)	374.3 (178.0)	332.8 (64.1)	335.9 (113.1)	300.0 (79.0)	325.6 (67.3)	299.9 (51.1)	**6.87/ .001/.07**	**6.46/ .012/.03**	2.38/ .09/.04
- SDRT (*M*, *SD*)	170.2 (151.0)	140.0 (85.0)	135.3 (105.6)	97.9 (60.6)	126.1 (106.3)	87.7 (54.5)	**8.15/ < .001/.08**	5.56/ .019/.02	0.95/ .39/.02

Note: Comparisons are based on variance analyses with normalized dependent variables, with age and sex as covariates and ASD, IQ and ASD by IQ as factors. Findings printed in bold are significant after correction for multiple testing using the False Discovery rate procedure with a q-value setting of .05 (correction for 16 variables, correction separately for both main effects and the interaction effect). MRT = Mean reaction time; SDRT = Standard deviation of reaction time.

### Procedure

The testing took place at Karakter Child and Adolescent Psychiatry University Centre Nijmegen (BOA project) or at schools (SPIDER project). Due to time constraints, not all tasks were administered to all children (see Table 3 for exact numbers, missing data ranged from 1% (Baseline speed) to 18% (Prosody). Drop-out analyses were run to examine if results were influenced by replacing missing data. Stimulants were discontinued for at least 24 hours before testing and non-stimulants according to their plasma half-life to allow for sufficient wash-out. Children were motivated with small breaks. The order of administration of the tasks was counterbalanced across participants.

### Statistical Analyses

All analyses were carried out in SPSS version 20. Reaction time measures were normally distributed, error measures showed a negatively skewed distribution. All measures were successfully normalized using a Van der Waerden transformation (SPSS version 20) [50]. This transformation handles the influence outliers may have on the data (by ranking them as (very) high or low within the normal distribution), thereby fulfilling the assumptions of variance analyses [51]. Some standardized variables were mirrored, so that higher scores indexed a better performance on all domains (faster performance, fewer errors). A composite score was calculated by averaging all z-scores.

In first instance, an ANCOVA was used with IQ (below/average/above) and diagnosis (ASD yes/no) as fixed factors and age and sex as covariates, and the composite score as dependent variable to determine whether overall there were main effects for IQ and ASD and an IQ by ASD interaction effect. Thereafter, to examine domain specific effects, speed and accuracy were used for each task separately as dependent measures in multivariate analysis of covariance (MANCOVA), again using IQ (below/average/above) and diagnosis (ASD yes/no) as fixed factors and age and sex as covariates. Sensitivity analyses using the same models were run with FSIQ as continuous variable instead of between-group variable. The effect of IQ was further investigated using polynomial contrasts testing linear and quadratic effects. Post-hoc t-tests were conducted comparing the IQ-matched ASD and control groups with each other. Correction for multiple testing was performed using the False Discovery Rate procedure.

## Results

Available data indicated that there was 80% power to detect main effects of small to moderate size (d > .4), but this level of power was only achieved for interaction effects that were at least substantial (d > .8). Therefore, for interpretation of interaction effects, visual inspection was used in addition to formal statistical analyses. Means and standard deviations of the unstandardized scores are presented in Table 3. Test statistics of the multivariate and univariate analyses, corrected for age and sex, based on the standardized scores are presented in Table 3 and described below. Age had a large, significant multivariate effect on cognitive functioning (*F* (2, 228) = 99.79, *p* < .001), sex did not (*F* (2, 228) = 0.77, *p* = .47). Analyses on raw and standardized data revealed similar results; drop-out analyses revealed similar results for analyses with and without replacement of missing data.

### Composite score

**Fig 1:**
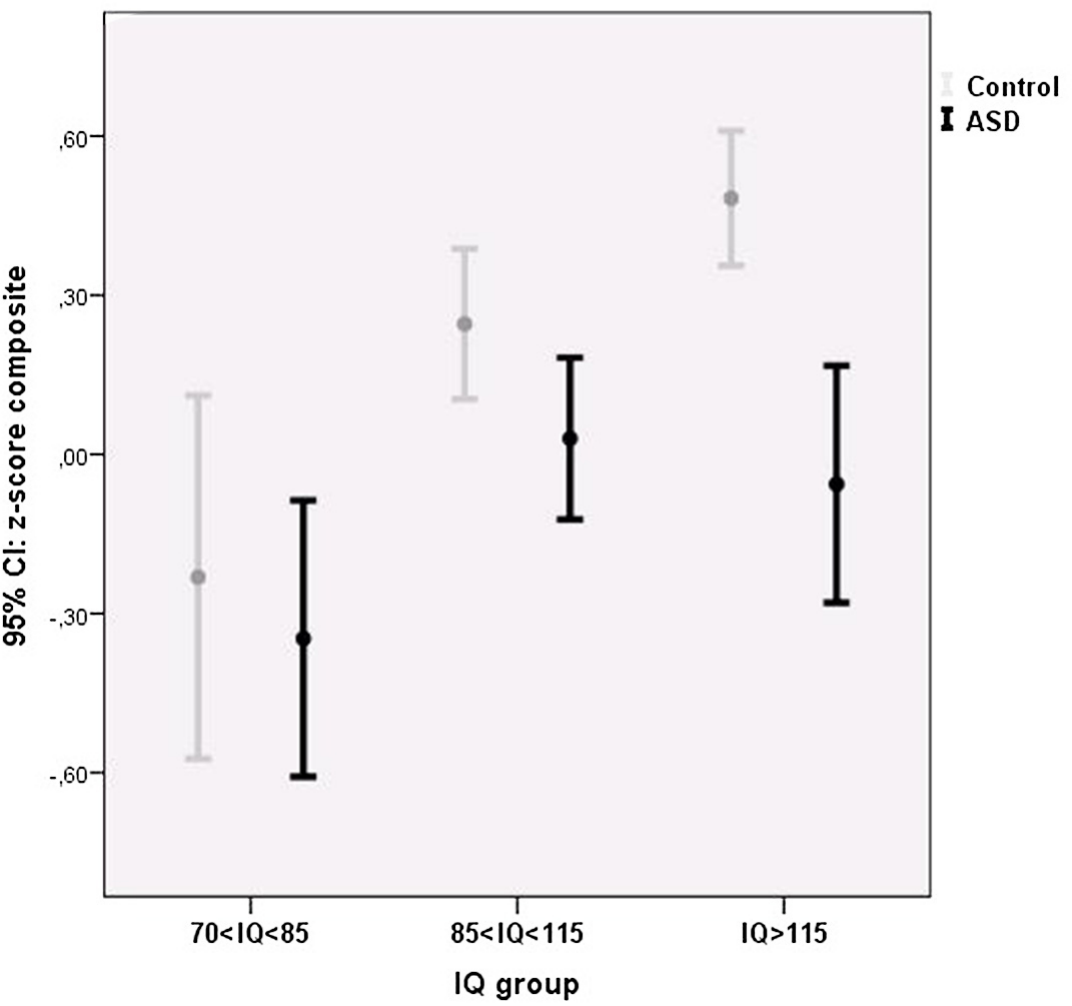
The effect of ASD and IQ on the cognitive composite score. Standardized z-score composed of the speed & accuracy measures of all task variables. Higher z-scores are indicative of better performance (faster performance and/or less errors).

A significant small main effect of ASD was present on the composite score, with participants with ASD overall performing poorer on the cognitive task battery than participants without ASD. A significant moderate effect of IQ was present, with higher IQ scores being related to better task performance. A small, trend significant diagnosis by IQ interaction was present (*p* = .12), that was significant when excluding the average IQ group from analyses (*p* = .04). Post-hoc comparisons indicated that participants with ASD and a higher IQ performed significantly poorer than the IQ matched controls (*t* = 4.50, *p* < .001), whereas this effect was smaller (albeit significant) for participants with ASD in the average IQ range (*t* = 2.09, *p* = .04) and absent in participants with ASD in the below average IQ range (*t* = 0.57, *p* .57) (Fig 1).

### Social cognition

**Fig 2:**
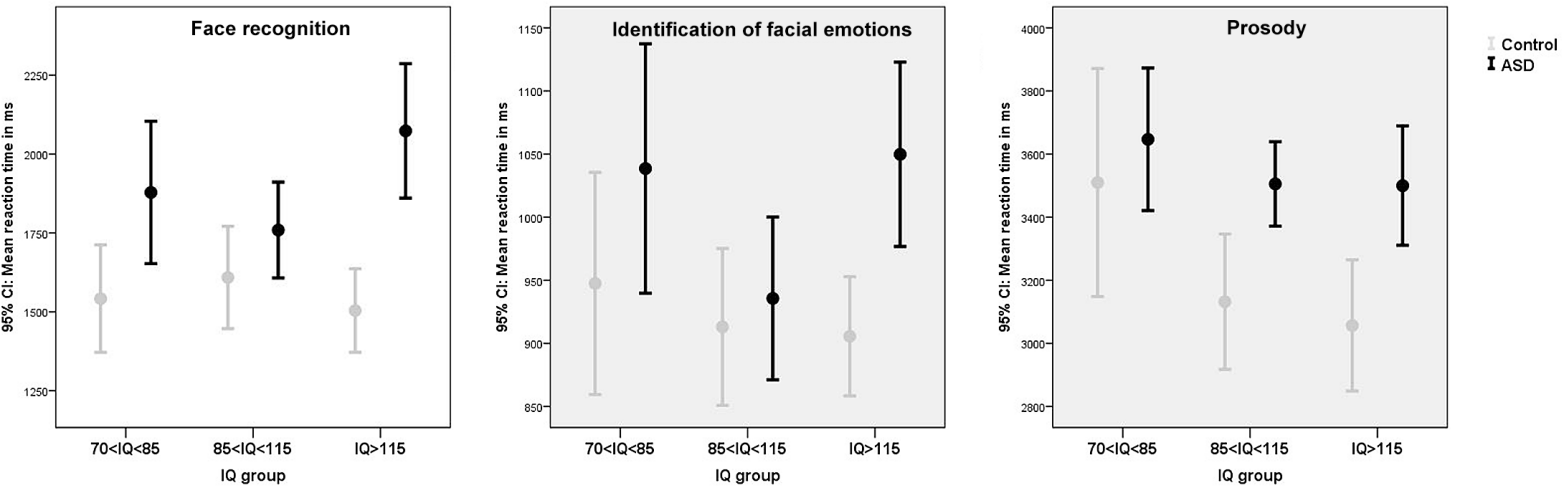
The effect of ASD and IQ on social cognition (face recognition, identification of facial emotions, prosody). Measures of speed are depicted. Please see S1 Fig for measures of accuracy.

A significant small to moderate main effect of ASD diagnosis was present for face recognition, in which children with ASD performed slower, but equally accurate, compared to controls. Similar effects were present on facial emotion recognition and affective prosody, albeit these did not survive correction for multiple testing. On all social cognition tasks, significant small to moderate effects of IQ were present (see Table 3 for test statistics). Polynomial contrasts indicated that quadratic contrasts best described the results with average IQ children performing most optimal in terms of speed and accuracy compared to below and above average IQ children (face recognition: speed Contrast Estimate [CE] for z-standardized scores = -.22, *p* = .008, accuracy CE = -.26, *p* = .002; facial emotion recognition: speed CE = -.27, *p* = .001, accuracy CE = -.16, *p* = .075; affective prosody: speed CE = -.22, *p* = .04, accuracy CE = -.31, *p* = .004). There were no significant or trend significant diagnosis by IQ interactions for any of the social cognition tasks even when IQ was treated as a continuous measure (all p's>.35) (Fig 2; S1 Fig).

### Executive functioning

**Fig 3:**
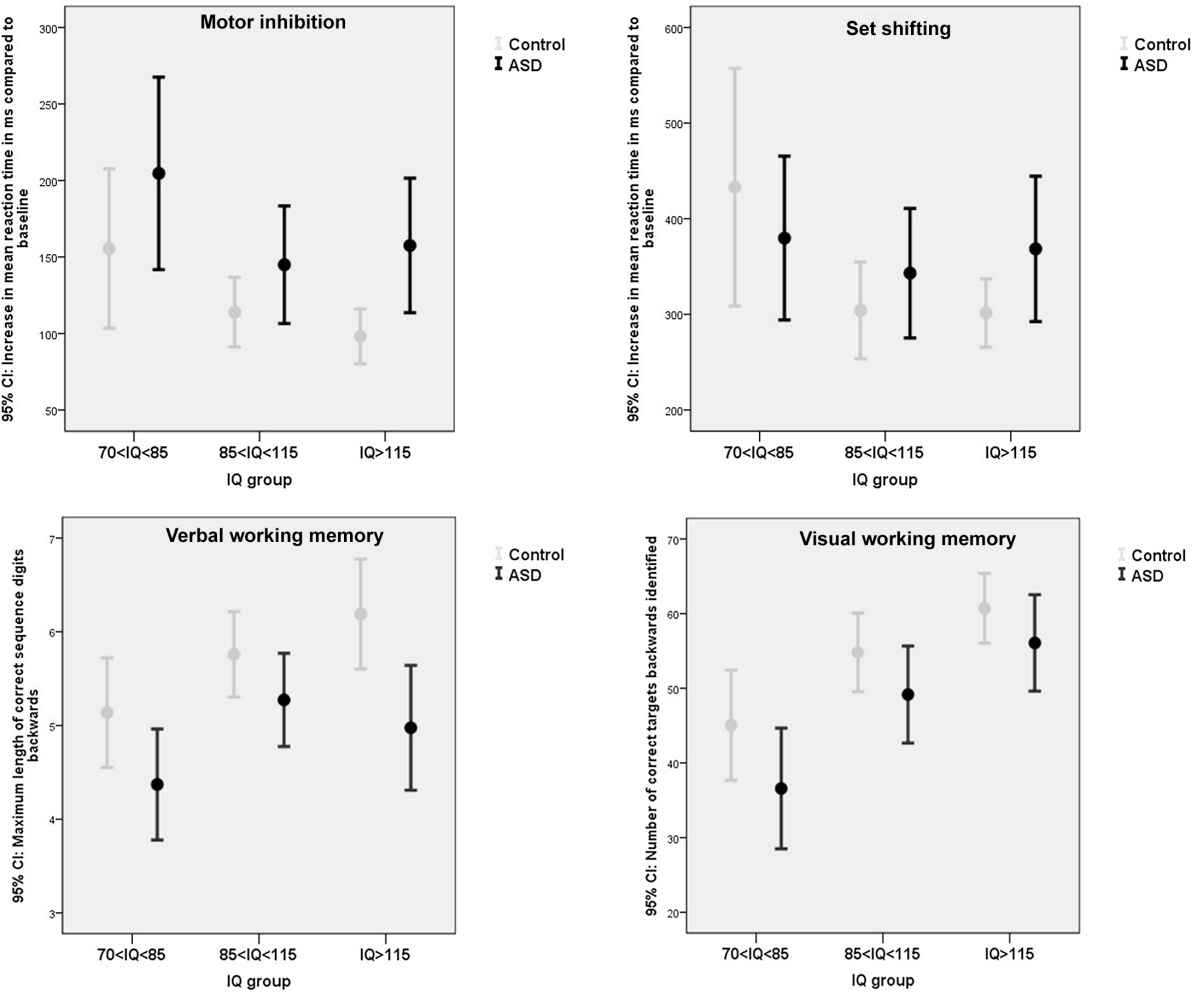
The effect of ASD and IQ on executive functioning (motor inhibition, set shifting, verbal working memory, visual working memory). Measures of speed are depicted for inhibition and set shifting. Please see S2 Fig for measures of accuracy.

A small to moderate effect of ASD diagnosis was present for verbal working memory and a small effect for inhibition, with control children outperforming children with ASD (see Table 3 for test statistics). No effect for cognitive flexibility was present and the group difference on visuo spatial working memory did not survive correction for multiple testing. Small to moderate significant effects of IQ were present for all tasks. Polynomial contrast indicated that linear terms best described the results (z-standardized scores: inhibition: speed CE = .40, *p* < .001, accuracy CE = .05, *p* = .65; cognitive flexibility: speed CE = .25, *p* = .03, accuracy CE = .22, *p* = .074; verbal working memory: CE = .30, *p* = .004; visuo-spatial working memory CE = .61, *p* < .001) with above average IQ children performing most optimal -and below average IQ children least optimal- in terms of speed and accuracy. There were no significant or trend significant diagnosis by IQ interactions for any of the social cognition tasks even when IQ was treated as a continuous measure (all p's>.39) (Fig 3; S2 Fig).

### Visual pattern recognition

**Fig 4:**
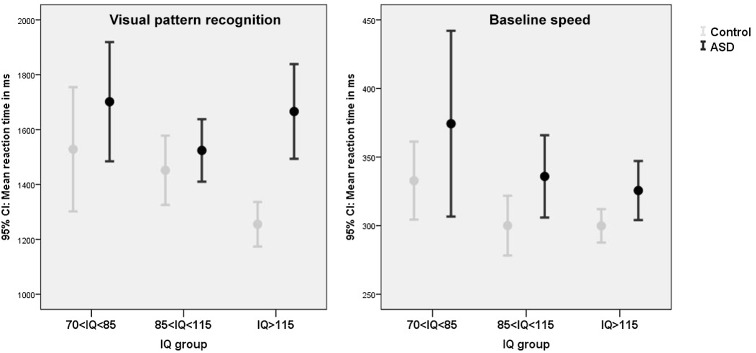
The effect of ASD and IQ on visual pattern recognition and baseline speed. Measures of speed are depicted. Please see S3 Fig for measures of accuracy (visual pattern recognition) and variability (baseline speed).

A trend significant effect of ASD diagnosis on visual pattern recognition was found, with ASD participants being somewhat slower than controls, but this effect did not survive correction for multiple testing (see Table 3 for test statistics). A significant effect of IQ emerged, which could best be described in linear terms (z-standardized scores: speed CE = .38, *p* < .001, accuracy CE = .60, *p* < .001) indicating above average IQ children performed most optimal -and below average IQ children least optimal- in terms of speed and accuracy. There were no significant or trend significant diagnosis by IQ interactions for any of the social cognition tasks even when IQ was treated as a continuous measure (all p's>.35) (Fig 4; S3 Fig).

### Basic processing speed

A significant small effect of ASD was present for basic processing speed, with ASD children being more variable in their reaction times. Small to moderate effects of IQ were present (see Table 3 for test statistics). Polynomial contrast indicated that speed could be described in linear (z-standardized scores: CE = .31, *p* < .001) and quadratic terms (CE = -.19, *p* = .017), and variability in linear terms (CE = .44, *p* < .001). Overall, above average IQ children were faster and less variable compared to below average IQ children. There were no significant or trend significant diagnosis by IQ interactions for any of the social cognition tasks even when IQ was treated as a continuous measure (all p's>.20) (Fig 4; S3 Fig).

## Discussion

The current study set out to examine the *absolute* and *relative* severity of cognitive deficits in participants with ASD in relation to IQ. The composite score revealed a trend significant IQ by ASD interaction, with above average intelligent participants with ASD performing *relatively* more poorly (*p* < .001) when compared to IQ matched controls (particularly visible for social cognition, visual pattern recognition and verbal working memory) in comparison to below average intelligent participants with ASD (*p* = .57). However, in *absolute* terms, participants with ASD and a below average IQ performed poorest, with robust main effects showing that ASD status and lower IQ negatively predicted cognitive performance. Only for social cognition, children with average IQs outperformed children with below and above average IQs.

The finding that above average intelligent individuals with ASD may be *relatively* most severely affected regarding cognitive skills, appeared mainly driven by effects on the domains of identification of facial identify and emotions, visual pattern recognition, and verbal working memory. It is difficult to identify a common denominator in these three measures, for identification of facial identify and emotions are measures of social cognition, visual pattern recognition is linked to non-social perceptual skills, and verbal working memory is considered at the core of executive functions. Particularly the finding on visual pattern recognition is unexpected since an information processing style favoring perception of details rather than global features has often been reported as a strength of individual with ASD [52, 53]. The results for social cognition concur with previous studies and clinical impressions of higher functioning ASD patients being most abnormal in their social receptiveness [54, 55] and may fit Baron-Cohen’s male-brain theory of autism, with higher-functioning patients exhibiting more systemizing and less empathizing skills [56]. These findings suggest that even though high IQ ASD individuals enjoy a certain protection from their higher IQ, they clearly demonstrate cognitive impairments. Their high IQ may make them appear cognitively rather similar to their healthy peers, despite our study as well as previous ones indicating they are not [57,58]. In other words, they are at risk that their social environment overestimates their capacities, which can contribute to the development of behavioural problems [59]. Further, despite their high IQ, they may lag behind in certain cognitive and information processing skills compared to their IQ matched peers. This puts these individuals at risk for being overestimated and calls for clinical attention, appropriate cognitive assessment and intervention. In any case, cognitive assessment and training of socio-cognitive skills [60–62] may be highly relevant to clinical and educational decision making in these higher functioning patients.

Conversely, even though in absolute terms ASD patients with below average IQs were clearly more impaired than ASD patients with average to above average IQs, the difference in cognitive functioning between participants with and without ASD on the lower end of the IQ spectrum were less pronounced. These findings tentatively suggest that intelligence may act as a moderator in the cognitive presentation of ASD, with qualitatively different cognitive processes affected in patients at the high and low end of the IQ spectrum. Or to put it simpler: ASD presents differently at different parts of the IQ spectrum. Clinically this may imply that cognitive assessment (and possibly training) of cognitive skills in below average intelligent children with ASD may be a less fruitful endeavour, since the cognitive characteristics appear less relevant in the clinical presentation of the disorder. However, this hypothesis needs firm replication in better powered studies, since our sample of below average intelligent participants was rather small. In any case, children in the lower end of the IQ continuum (with or without ASD) performed in absolute terms clearly weaker than (above) average intelligent children, underlying their need for guidance and environmental support to optimize their social, school and occupational outcomes.

Compared to the above outlined diagnosis by IQ interaction effect, main effects of ASD and IQ were clearly more robust, with ASD patients performing overall poorer than controls regarding social cognition (face recognition, and to a lesser extent facial emotion recognition and prosody), executive functions (verbal working memory and to a lesser extent inhibition and visual-spatial working memory), visual pattern recognition and BPS. These findings are in line with previous studies [34–36, 38–40]. Group differences were overall small to moderate and mainly described in terms of decreased speed or memory load, instead of increased error rates. Importantly however, cognitive deficits were not generalized across all domains (particularly set shifting was completely normal in ASD participants across the IQ range, in line with a recent review [63] and were overall of small to moderate size at best, suggesting cognitive deficits not to be the core or sole cause of the substantial daily life impairments patients with ASD face. As expected [20, 64,65], above average IQ scores generally predict optimal performance on a wide range of cognitive domains. However, on the social domain the effects of IQ on performance was best described in quadratic–and not linear- terms, with average IQ children performing most optimal in terms of accuracy and speed. This concurs with earlier findings [66] and may suggest that the mind of individuals with above average IQs may be more directed towards dealing with abstract concepts and ideas than to an accurate perception and interpretation of the immediate social environment.

Several limitations are to be noted. First of all, our study included a relatively small sample size of below average IQ children, particularly controls. This may have hampered the detection of multivariate diagnosis by IQ interactions. Replication of these effects in better powered samples is warranted. Second, we were not able to completely match all four groups regarding sex. Analyses were therefore corrected for sex. Third, IQ estimates were based on only four subtests (Similarities, Block Design, Picture Completion and Vocabulary) and the discretization of IQ to form below, average and above average IQ groups. In addition, Block Design seems the best performal subtest in particularly children with ASD [63], suggesting PIQ may have been overestimated in the ASD group based on this short form. However, these specific subtests are known to correlate strongly (>.90) with the Full scale IQ and discretization provides illustratable and clinically meaningful results. Also, every effort was made to match the ASD and control groups regarding every aspect of IQ performance on these tasks (FSIQ, VIQ, PIQ, VIQ-PIQ discrepancy), making it unlikely that the administration of an IQ sub-battery explains the overall absence of diagnosis by IQ interaction effects. Fourth, SCQ scores were lower in the low and average IQ ASD group than in the above average IQ group. This may suggest IQ and severity of ASD were confounded. Alternatively, it may also be inherent to ASD in above average IQ children, with ASD symptoms being masked by the overall good verbal skills. In any case, our results suggest that even though SCQ scores were lower in above average IQ ASD children, they still suffer from similar cognitive deficits as average or below average IQ ASD children. A final limitation if the lack of including children with IQs <70. The reason for not doing so, is that the cognitive task battery was too complicated for children with IQs in this range. Future research needs to indicate whether the conclusions of our study also extrapolate towards this range of intellectual functioning, or that ASD with IQs< 70 is characterized by qualitatively different cognitive profiles compared to ASD in combination with IQs>70.

In conclusion, in *relative* terms, cognitive deficits appear somewhat more severe in individuals with ASD and above average IQs compared to the below average IQ patients with ASD. Even though high IQ ASD individuals enjoy a certain protection from their higher IQ, they clearly demonstrate cognitive impairments that may be targeted in clinical assessment and treatment. Conversely, even though in *absolute* terms ASD patients with below average IQs were clearly more impaired than ASD patients with average to above average IQs, the difference in cognitive functioning between participants with and without ASD on the lower end of the IQ spectrum were less pronounced. Clinically this may imply that cognitive assessment and training of cognitive skills in below average intelligent children with ASD may be a less fruitful endeavour. These findings tentatively suggest that intelligence may act as a moderator in the cognitive presentation of ASD, with qualitatively different cognitive processes affected in patients at the high and low end of the IQ spectrum.

## Supporting Information

S1 Data Raw data reported in the manuscript. (SAV)

S1 Fig The effect of ASD and IQ on social cognition. (TIF)

S2 Fig The effect of ASD and IQ on executive functioning. (TIF)

S3 Fig The effect of ASD and IQ on visual pattern recognition and baseline speed. (TIF)
