# Supplementary figures and images for: Intelligence May Moderate the Cognitive Profile of Patients with ASD
Source: PLoS One. 2015 Oct 7;10(10):e0138698. doi: 10.1371/journal.pone.0138698 (PMC4596821; doi:10.1371/journal.pone.0138698)

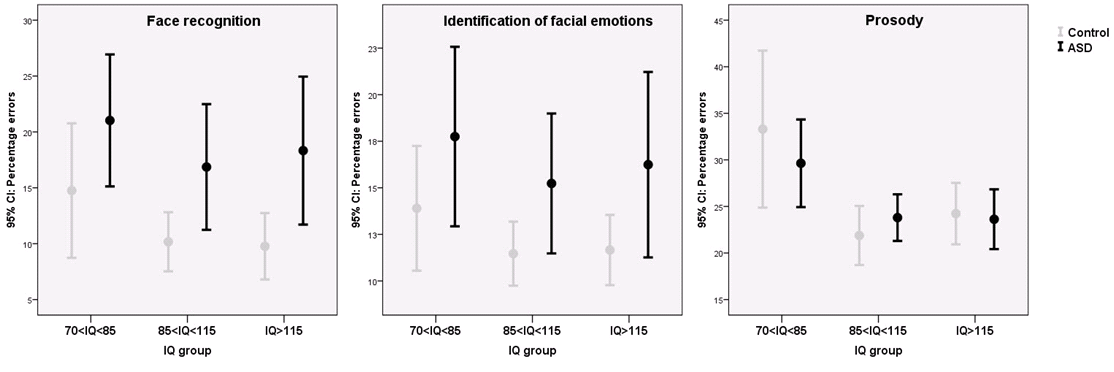

Supplement: S1 Fig — (TIF) [file pone.0138698.s002.tif]

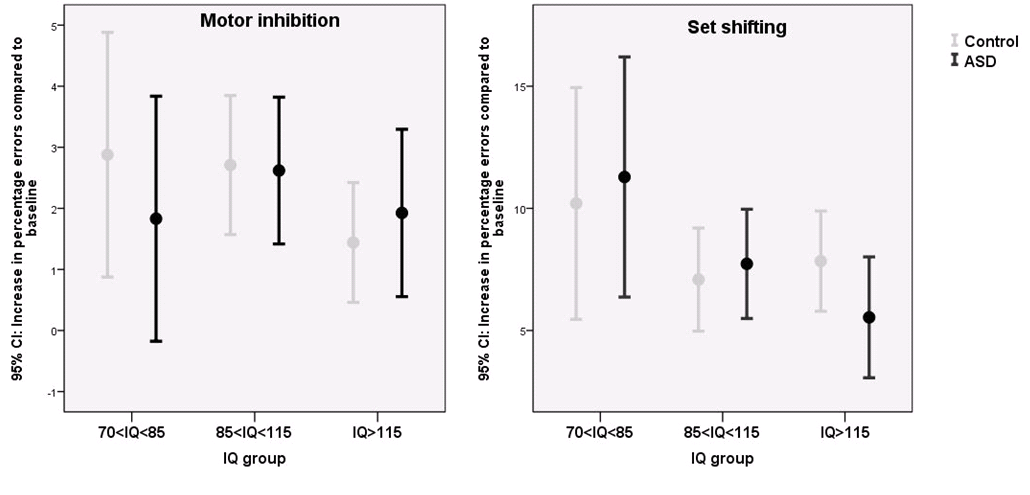

Supplement: S2 Fig — (TIF) [file pone.0138698.s003.tif]

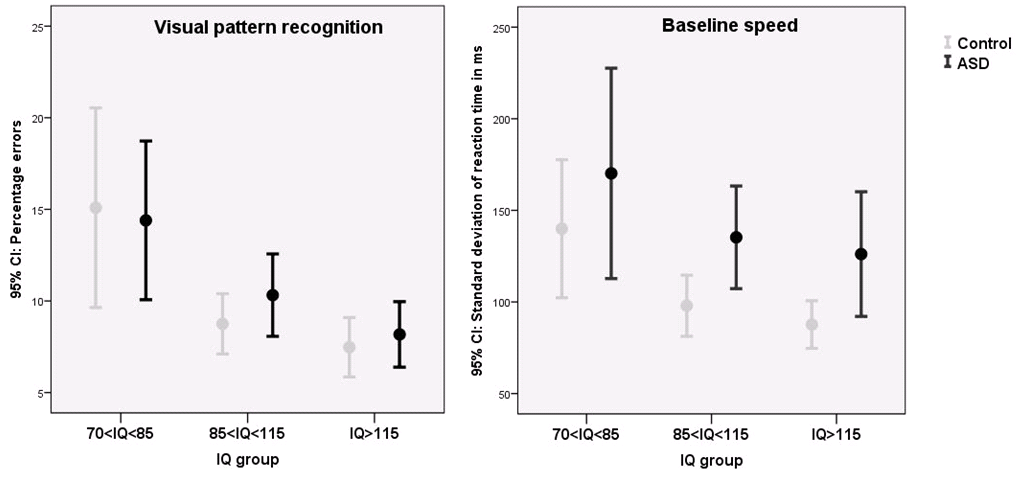

Supplement: S3 Fig — (TIF) [file pone.0138698.s004.tif]
